# Synthesis and Biological Evaluation of Novel ^99m^Tc(CO)_3_-Labeled Thymidine Analogs as Potential Probes for Tumor Proliferation Imaging

**DOI:** 10.3390/molecules21040510

**Published:** 2016-04-19

**Authors:** Xiaojiang Duan, Teli Liu, Yichun Zhang, Junbo Zhang

**Affiliations:** Key Laboratory of Radiopharmaceuticals (Beijing Normal University), Ministry of Education, College of Chemistry, Beijing Normal University, Beijing 100875, China; duanxj10@gmail.com (X.D.); liuteli123321@163.com (T.L.); zhangyichunkeke@163.com (Y.Z.)

**Keywords:** thymidine analogs, click chemistry, [^99m^Tc(CO)_3_]^+^ core, tumor proliferation imaging, macrocyclic triamine

## Abstract

Achieving a ^99m^Tc labeled thymidine radiotracer for single photon emission tomography (SPECT) is considered to be of interest. In this study, four novel thymidine analogs, **6a**, **6b**, **6c** and **6d**, were successfully synthesized via “click reaction” route and then radiolabeled using a [^99m^Tc(CO)_3_]^+^ core to prepare the corresponding ^99m^Tc(CO)_3_ complexes in high yields. These complexes were hydrophilic and had good *in vitro* stability. Biodistribution of these complexes in mice bearing S180 tumors showed that all of them exhibited accumulation in the tumors, suggesting that they would be potential tumor imaging agents.

## 1. Introduction

The sugar-based PET (Positron Emission Tomography) radiotracer 2-[^18^F]fluoro-2-deoxy-d-glucose (^18^F-FDG), the gold standard tracer for tumor detection and staging clinically, still has some limitations, like producing false-positive or negative results, low image contrast in brain tumor diagnosis and poor differentiation of tumor from inflammation [1,2]. Continued proliferation is one of the hallmarks of cancer [3], and DNA synthesis is the most direct metabolic process relating to the cell proliferation [4]. So many investigators have developed various radiolabeled DNA precursors, especially labeled thymidine and thymidine analogs. These radiolabeled tracers with positron emitters, such as ^11^C-labeled nucleoside thymidine [5], 3′-deoxy-3′-[^18^F]fluorothymidine (^18^F-FLT) [6,7,8,9,10], 2′-fluoro-5-[^11^C]-methyl-1-beta-d-arabinofuranosyluracil (^11^C-FMAU) [11] and 2′-[^18^F]fluoro-5-methyl-1-beta-d-arabinofuranosyluracil (^18^F-FMAU) [12,13,14,15], are all substrates of the human thymidine kinase 1 (TK1). ^18^F-FLT, in particular, is an effective tracer of tumor proliferation, and is a specific and clinically relevant prognostic predictor in the treatment of cancer [16]. However, an expensive cyclotron is essential in the production of these radionuclides, restricting their wide use in clinical practice. By comparison, ^99m^Tc can be obtained at a reasonable cost, which makes it readily available and affordable. The availability of a generator and kit chemistry to prepare ^99m^Tc based radiotracers may have a significant impact on nuclear medicine. Thus, using ^99m^Tc to label thymidine analogs is the focus of the ongoing research. To date, several ^99m^Tc labeled thymidine analogs have been reported [17,18,19,20,21,22,23]. However, none of them showed the ideal properties. The preparation of novel ^99m^Tc labeled thymidine analogs is still considered to be of great necessity and a considerable challenge.

Recently, the [^99m^Tc(CO)_3_]^+^ complex has attracted significant attention due to its ease of preparation, readily substituted water molecules of the precursor *fac*-[^99m^Tc(CO)_3_(H_2_O)_3_]^+^ by a variety of tridentate ligands, small size, and inertness [24]. Struthers *et al.* had synthesized many thymidine analogs labeled with technetium and rhenium tri-carbonyl as the substrates of TK1 [25,26]. Among different tridentate ligands, a macrocyclic triamine compound, such as 1,5,9-triazacyclododecane (12N3), is a suitable ligand for radiolabeling with the [^99m^Tc(CO)_3_]^+^ core [27]. The copper-(I)-catalyzed “click chemistry” by reacting azide with terminal alkyne to form 1,2,3-triazoles (for the trizole’s stability in biocircumstances) is used in the area of bioconjugation reactions. It has played a vital role in the synthesis of compounds, as well as in the field of radiopharmaceuticals and molecular imaging [28,29,30].

In this study, four novel thymidine analogs, **6a**, **6b**, **6c** and **6d**, were successfully synthesized via “click chemistry” route and then radiolabeled using [^99m^Tc(CO)_3_]^+^ core to prepare the corresponding ^99m^Tc(CO)_3_ complexes. The partition coefficient, stability *in vitro*, cell uptake, and biodistribution in mice were also evaluated.

## 2. Results and Discussion

### 2.1. Chemistry and Radiolabeling

**Scheme 1:**
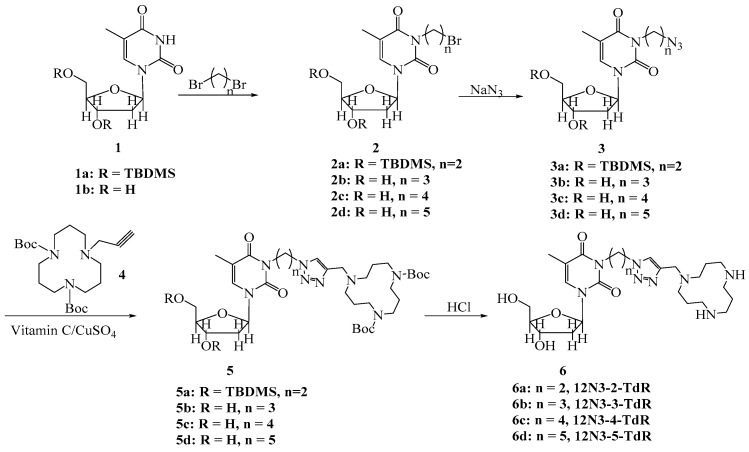
The syntheses of **6a**–**d**.

**Scheme 2:**
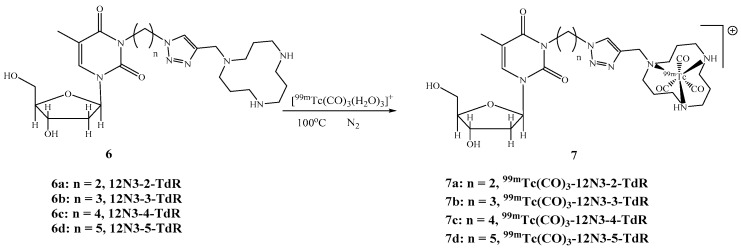
The radiochemical syntheses of **7a**–**d** and their proposed structures.

**Figure 1:**
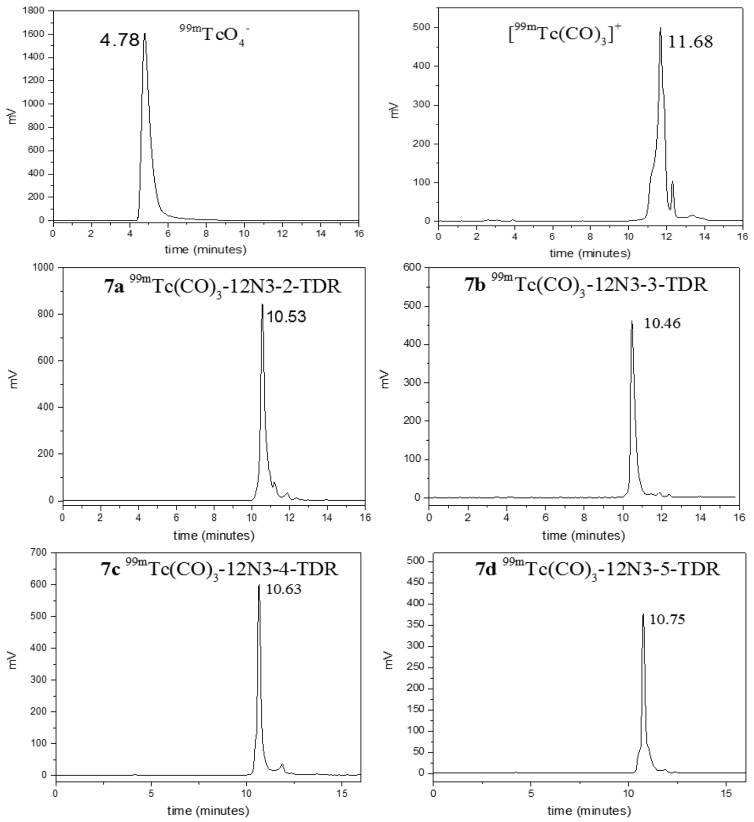
HPLC chromatograms of ^99m^TcO_4_^−^ (t_R_ = 4.78 min), [^99m^Tc(CO)_3_]^+^ (t_R_ = 11.68 min), **7a** (t_R_ = 10.53 min), **7b** (t_R_ =10.46 min), **7c** (t_R_ = 10.63 min) and **7d** (t_R_ = 10.75 min).

Compounds **6a**, **6b**, **6c** and **6d** were synthesized by multi-step reactions from the starting materials thymidine via “click” reaction. The reaction equations were shown in Scheme 1. They were identified by ^1^H-NMR, ^13^C-NMR, ESI-MS (Electrospray Ionization Mass Spectrometry), HRMS (High Resolution Mass Spectrometry) and the results agreed well with the expected chemical structures. The final products were suitable tridentate precursors for the [^99m^Tc(CO)_3_]^+^ core (Scheme 2). Compounds **7a**, **7b**, **7c** and **7d** were obtained with high radiochemical purity (Figure 1).

According to the results of HPLC analysis, the radiochemical purities of the complexes amount to more than 90%.

Suzuki *et al.* [27] reported that ^99m^Tc(CO)_3_-12N3 was not stable at an elevated temperature. Recently, we have discovered that the ^99m^Tc(CO)_3_(H_2_O)_3_^+^ could be reconverted to ^99m^TcO_4_^−^ at 100 °C without the protection of nitrogen. Moreover, **6a**, **6b**, **6c** and **6d** ligands should react with ^99m^Tc(CO)_3_(H_2_O)_3_^+^ in order to obtain the desired products under nitrogen.

### 2.2. Stability and Partition Coefficients

The stability of the complexes was assayed by measuring the radiochemical purity by HPLC (High Performance Liquid Chromatography). The complex was stable over 6 h in the reaction mixture at room temperature (HPLC chromatograms can be found in the Supplementary Materials Figure S1). Only a little decomposition of the complexes was observed in the mouse serum at 37 °C for 6 h (HPLC chromatograms can be found in the Supplementary Materials Figure S2), suggesting that they had good *in vitro* stability.

The log *p* values of **7a**, **7b**, **7c** and **7d** were −1.16 ± 0.01, −0.97 ± 0.01, −1.09 ± 0.01 and −1.13 ± 0.01, respectively. The results suggested that all of them were hydrophilic.

### 2.3. In Vitro Cell Experiments

**Figure 2:**
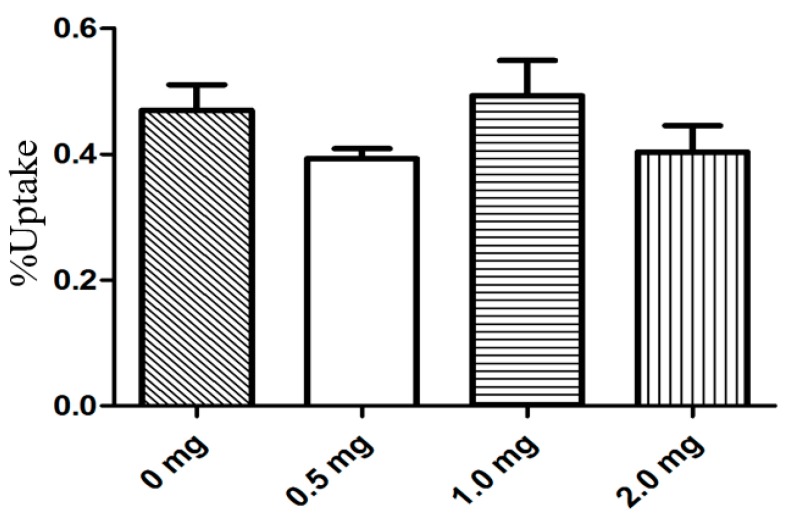
*In vitro* cell uptake of **7d** when different amount thymidine was administered.

*In vitro* cell uptake of **7d** using S180 cells showed that there was no significant difference (*p* > 0.05) between control and blocking groups (Figure 2). The results indicated the tumor uptake of **7d** was related to a nonspecific diffusion. The complex possibly exhibits the overall positive charge, thus making it pass the tumor cell membrane.

### 2.4. Biodistribution Study

The results of biodistributions of **7a**, **7b**, **7c** and **7d** in tumor-bearing mice were shown in Table 1, Table 2, Table 3 and Table 4, respectively. At 30 min post-injection, the tumor uptakes of **7a**, **7b**, **7c** and **7d** were 2.16% ± 0.59%, 1.28% ± 0.46%, 1.57% ± 0.31% and 1.90% ± 0.58%ID/g. At 360 min post-injection, the tumor uptakes of the complexes were 0.55% ± 0.14%, 0.46% ± 0.08%, 0.40% ± 0.06% and 0.36% ± 0.16%ID/g. These results indicated that all of them exhibited an accumulation in the tumor. At 30 min post-injection, **7d** exhibited a higher tumor/muscle ratio (3.56) and **7a** had the highest tumor uptake (2.16% ± 0.59%ID/g). It was found that the tumor/muscle ratio at 30 min post-injection increased with the increase of the carbon chain length between thymidine and the 12N3. Desbouis *et al.* [25] had discovered that a long spacer between the thymidine and organometallic core would improve the ability of the complexes to be accommodated in the binding site. The discovery might be the reason for the increasing uptake ratio of tumor to muscle.

Compared with ^99m^Tc-12N3 [27], the complexes **7a**, **7b**, **7c** and **7d** were also mainly accumulated in the excretory organs such as liver, kidneys and intestines, suggesting that the major route of excretion was renal and hepatobiliary. The bone uptakes of the four complexes were more than that of ^99m^Tc-12N3, which demonstrates a selective uptake in the marrow, a tissue with a large number of proliferative cells [6]. Low uptake in the stomach and thyroid was indicative of *in vivo* stability of these complexes.

## 3. Experimental Section

### 3.1. General

All chemical reagents were purchased from commercial sources and used without any further purification. ^99^Mo/^99m^Tc generator was obtained from the China Institute of Atomic Energy (CIAE). NMR spectra were obtained on a 400 MHz Bruker Avance 500 spectrometer (Bruker, Billerica, MA, USA). ESI-MS spectra were obtained on a LC-MS Shimadzu 2010 series (Shimadzu, Kyoto, Japan). HRMS spectra were obtained on a AB SCIEX TripleTOF™ 5600(AB Sciex, Concord, ON, Canada). HPLC analysis was performed on a Waters 600 binary HPLC pump (Waters, Milford, MA, USA) and a Waters 2487 UV absorbance dual λ detector (Waters, Milford, MA, USA) with a reversed-phase column (Kromaisl C18, 250 mm × 4.6 mm) (AkzoNobel, Bohus, Sweden). Murine sarcoma S180 cell line was obtained from Peking University Health Science Center (Beijing, China).

### 3.2. Synthesis

The syntheses of 12N3-2-TdR (**6a**), 12N3-3-TdR (**6b**), 12N3-4-TdR (**6c**) and 12N3-5-TdR (**6d**) are depicted in Scheme 1. Compound **1a** was synthesized according to the literature [31]. Compound **4** was synthesized according to the literature [32].

*3-(2-Bromoethyl)-1-((2R,4S,5R)-4-((tert-butyldimethylsilyl)oxy)-5-(((tert butyldimethylsilyl)oxy)methyl)tetrahydrofuran-2-yl)-5-methylpyrimidine-2,4(1H,3H)-dione* (**2a**). Compound **2a** was prepared according to the literature [25]. Namely, compound **1a** (1 g, 2.13 mmol) was added into DMF (*N*,*N*-Dimethylformamide) (10 mL). Cs_2_CO_3_ (2.08 g, 6.39 mmol) was added and the mixture was stirred for 5 min at room temperature. After an addition of 1, 2-dibromoethane (11.94 g, 63.8 mmol), the reaction was stirred for another 2 h at room temperature, and was followed by TLC (Thin Layer Chromatography). Solvent was evaporated under a reduced pressure and the crude product was purified by column chromatography with CH_2_Cl_2_ to give **2a** as white solid (900 mg, yield 73%). ^1^H-NMR (400 MHz, CDCl_3_): δ 7.47 (d, *J* = 1.3 Hz, 1H), 6.34 (dd, *J* = 8.0, 5.8 Hz, 1H), 4.44–4.25 (m, 3H), 3.93 (q, *J* = 2.5 Hz, 1H), 3.80 (ddd, *J* = 42.1, 11.4, 2.6 Hz, 2H), 3.52 (t, *J* = 7.3 Hz, 2H), 2.25 (ddd, *J* = 13.1, 5.8, 2.6 Hz, 1H), 2.03–1.94 (m, 1H), 1.91 (s, 3H), 0.89 (d, *J* = 12.7 Hz, 18H), 0.10 (s, 6H), 0.07 (s, 3H), 0.06 (s, 3H). ^13^C-NMR (100 MHz, CDCl_3_): δ 163.0, 150.6, 133.8, 110.0, 87.9, 85.6, 72.3, 63.0, 42.1, 41.4, 27.2, 26.0, 25.8, 18.4, 18.0, 13.2, −4.6, −4.8, −5.4, −5.4. ESI-MS (*m*/*s*): 578.9 (calc. 579.2 [M + 3H^+^]). HRMS(*m*/*s*): 577.2122 [C_24_H_45_N_2_O_5_Si_2_Br]H^+^ (calc. 577.2123).

*3-(3-Bromopropyl)-1-((2R,4S,5R)-4-hydroxy-5-(hydroxymethyl)tetrahydrofuran-2-yl)-5 methylpyrimidine-2,4(1H,3H)-dione* (**2b**). Compound **1b** (1.9 g, 8 mmol) was added in DMF (30 mL). K_2_CO_3_ (3.3 g, 24 mmol) was added and the mixture was stirred for 5 min at room temperature. After an addition of 1,3-dibromopropane (1.62 g, 8 mmol), the reaction was stirred for another 18 h at room temperature and was followed by TLC. Solvent was evaporated under a reduced pressure and the crude product was purified by column chromatography with EtOAc to give **2b** as white solid (1.9 g, yield 67%). ^1^H-NMR (400 MHz, CDCl_3_): δ 7.46 (d, *J* = 1.3 Hz, 1H), 6.21 (t, *J* = 6.7 Hz, 1H), 4.54 (td, *J* = 5.1, 3.6 Hz, 1H), 4.09–4.02 (m, 2H), 3.99 (q, *J* = 3.3 Hz, 1H), 3.85 (ddd, *J* = 42.2, 11.4, 2.6 Hz, 2H), 3.40 (t, *J* = 6.9 Hz, 2H), 3.14 (s, 2H), 2.37–2.30 (m, 2H), 2.24–2.11 (m, 2H), 1.90 (d, *J* = 1.0 Hz, 3H). ^13^C-NMR (100 MHz, MeOD): δ 165.4, 152.4, 136.5, 110.7, 88.9, 87.2, 72.0, 62.8, 41.4, 41.3, 32.1, 31.1, 13.1. ESI-MS (*m*/*s*): 385.0 (calc. 385.0 [M + Na^+^]). HRMS (*m*/*s*): 363.0548 [C_13_H_19_N_2_O_5_Br]H^+^ (calc. 363.0550).

*3-(4-Bromobutyl)-1-((2R,4S,5R)-4-hydroxy-5-(hydroxymethyl)tetrahydrofuran-2-yl)-5-methylpyrimidine-2,4(1H,3H)-dione* (**2c**). Compound **1b** (300 mg, 1.23 mmol) was added in DMF (10 mL). K_2_CO_3_ (512 mg, 3.75 mmol) was added and the mixture was stirred for 5 min at room temperature. After an addition of 1,4-dibromobutane (267 mg, 1.23 mmol), the reaction was stirred for another 7 h at room temperature, and was followed by TLC. Solvent was evaporated under a reduced pressure and the crude product was purified by column chromatography with EtOAc to give **2c** as white solid (310 mg, yield 67%). ^1^H-NMR (400 MHz, CDCl_3_): δ 7.42 (d, *J* = 1.3 Hz, 1H), 6.21 (t, *J* = 6.8 Hz, 1H), 4.55 (dt, *J* = 6.2, 3.8 Hz, 1H), 4.00 (q, *J* = 3.2 Hz, 1H), 3.98–3.77 (m, 4H), 3.42 (t, *J* = 6.6 Hz, 2H), 3.04 (s, 2H), 2.42–2.27 (m, 2H), 1.96–1.82 (m, 5H), 1.76 (td, *J* = 8.1, 6.7 Hz, 2H). ^13^C-NMR (100 MHz, CDCl_3_): δ 163.5, 150.8, 134.8, 110.0, 87.0, 86.2, 71.1, 62.1, 40.3, 40.2, 33.3, 29.9, 26.2, 13.2. ESI-MS (*m*/*s*): 379.0 (calc. 379.1 [M + 3H^+^]). HRMS (*m*/*s*): 377.0710 [C_14_H_21_N_2_O_5_Br]H^+^ (calc. 377.0706).

*3-(5-Bromopentyl)-1-((2R,4S,5R)-4-hydroxy-5-(hydroxymethyl)tetrahydrofuran-2-yl)-5-methylpyrimidine-2,4(1H,3H)-dione* (**2d**). Compound **1b** (300 mg, 1.23 mmol) was added in DMF (10 mL). K_2_CO_3_ (512 mg, 3.75 mmol) was added and the mixture was stirred for 5 min at room temperature. After an addition of 1,5-dibromopentane (285 mg, 1.23 mmol), the reaction was stirred for another 7 h at room temperature, and was followed by TLC. Solvent was evaporated under a reduced pressure and the crude product was purified by column chromatography with EtOAc to give **2d** as white solid (300 mg, yield 62%). ^1^H-NMR (400 MHz, CDCl_3_): δ 7.40 (d, *J* = 1.4 Hz, 1H), 6.20 (t, *J* = 6.8 Hz, 1H), 4.55 (dt, *J* = 6.2, 3.8 Hz, 1H), 4.00 (q, *J* = 3.3 Hz, 1H), 3.95–3.76 (m, 4H), 3.39 (t, *J* = 6.7 Hz, 2H), 3.02 (s, 2H), 2.42–2.26 (m, 2H), 1.94–1.81 (m, 5H), 1.69–1.56 (m, 2H), 1.47 (tt, *J* = 10.2, 4.1 Hz, 2H). ^13^C-NMR (100 MHz, CDCl_3_): δ 163.5, 150.9, 134.7, 110.0, 87.1, 86.4, 71.2, 62.2, 41.0, 40.3, 33.7, 32.2, 26.6, 25.34, 13.3. ESI-MS (*m*/*s*): 392.9 (calc. 393.0 [M + 3H^+^]). HRMS (*m*/*s*): 391.0865 [C_15_H_23_N_2_O_5_Br]H^+^ (calc. 391.0863).

*3-(2-Azidoethyl)-1-((2R,4S,5R)-4-((tert-butyldimethylsilyl)oxy)-5-(((tert butyldimethylsilyl)oxy)methyl)tetrahydrofuran-2-yl)-5-methylpyrimidine-2,4(1H,3H)-dione* (**3a**). Compound **3a** was prepared according to the literature [33]. Namely, compound **2a** (1.1 g, 1.91 mmol) and NaN_3_ (1.24 g, 19.1 mmol) were added into CH_3_CN (20 mL). The mixture was refluxed for 16 h and was followed by TLC. The solvent was evaporated under a reduced pressure and the crude product was purified by column chromatography with CH_2_Cl_2_ to give **3a** as white solid (800 mg, yield 78%). ^1^H-NMR (400 MHz,CDCl_3_): δ 7.48 (d, *J* = 1.3 Hz, 1H), 6.35 (dd, *J* = 7.9, 5.8 Hz, 1H), 4.39 (dt, *J* = 5.6, 2.6 Hz, 1H), 4.19 (td, *J* = 6.3, 2.1 Hz, 2H), 3.94 (q, *J* = 2.6 Hz, 1H), 3.81 (ddd, *J* = 42.4, 11.4, 2.6 Hz, 2H), 3.53 (t, *J* = 6.3 Hz, 3H), 2.26 (ddd, *J* = 13.2, 5.8, 2.6 Hz, 1H), 1.99 (ddd, *J* = 13.5, 7.9, 6.0 Hz, 1H), 1.93 (d, *J* = 1.1 Hz, 3H), 0.90 (d, *J* = 13.0 Hz, 18H), 0.11 (s, 3H), 0.10 (s, 3H), 0.08 (s, 3H), 0.07 (s, 3H). ^13^C-NMR (100 MHz, CDCl_3_): δ 163.0, 150.6, 133.7, 110.0, 87.7, 85.4, 72.1, 62.8, 48.2, 41.3, 39.6, 25.8, 25.6, 18.2, 17.8, 13.0, −4.8, −5.0, −5.6, −5.6. ESI-MS (*m*/*s*): 540.1 (calc. 540.3 [M + H^+^]). HRMS (*m*/*s*): 540.3033 [C_24_H_45_N_5_O_5_Si_2_]H^+^ (calc. 540.3032).

*3-(3-Azidopropyl)-1-((2R,4S,5R)-4-hydroxy-5-(hydroxymethyl)tetrahydrofuran-2-yl)-5-methylpyrimidine-2,4(1H,3H)-dione* (**3b**). Compound **2b** (1 g, 2.75 mmol) and NaN_3_ (1.79 g, 27.5 mmol) were added into CH_3_CN (30 mL). The mixture was refluxed for 8 h and was followed by TLC. The solvent was evaporated under a reduced pressure and the crude product was purified by column chromatography with EtOAc to give **3b** as colorless oil (570 mg, yield 63%). ^1^H-NMR (400 MHz, CDCl_3_): δ 7.42 (d, *J* = 1.3 Hz, 1H), 6.20 (t, *J* = 6.8 Hz, 1H), 4.56 (dt, *J* = 6.2, 3.6 Hz, 1H), 4.05–3.97 (m, 3H), 3.85 (ddd, *J* = 42.3, 11.4, 2.6 Hz, 2H), 3.34 (t, *J* = 6.8 Hz, 2H), 2.94 (s, 2H), 2.43–2.26 (m, 2H), 1.96–1.83 (m, 5H). ^13^C-NMR (100 MHz, CDCl_3_): δ 163.7, 151.0, 135.0, 110.3, 87.3, 86.8, 71.5, 62.4, 49.3, 40.2, 39.1, 27.1, 13.4. ESI-MS (*m*/*s*): 326.0 (calc. 326.3 [M + H^+^]). HRMS (*m*/*s*): 326.1461 [C_13_H_19_N_5_O_5_]H^+^ (calc. 326.1458).

*3-(4-Azidobutyl)-1-((2R,4S,5R)-4-hydroxy-5-(hydroxymethyl)tetrahydrofuran-2-yl)-5-methylpyrimidine-2,4(1H,3H)-dione* (**3c**). Compound **2c** (310 mg, 0.825 mmol) and NaN_3_ (536 mg, 8.24 mmol) were added into CH_3_CN (10 mL). The mixture was refluxed for 5 h and was followed by TLC. The solvent was evaporated under a reduced pressure and the crude product was purified by column chromatography with EtOAc to give **3c** as colorless oil (180 mg, yield 64%). ^1^H-NMR (400 MHz, CDCl_3_): δ 7.33 (d, *J* = 1.2 Hz, 1H), 6.18 (t, *J* = 6.9 Hz, 1H), 4.61 (dt, *J* = 6.8, 3.6 Hz, 1H), 4.02 (q, *J* = 3.1 Hz, 1H), 3.99–3.79 (m, 4H), 3.31 (t, *J* = 6.7 Hz, 2H), 2.45 (dt, *J* = 13.8, 6.9 Hz, 2H), 2.31 (ddd, *J* = 13.7, 6.4, 3.6 Hz, 1H), 2.13 (s, 1H), 1.93 (d, *J* = 1.2 Hz, 3H), 1.76–1.57 (m,4H). ^13^C-NMR (100 MHz, CDCl_3_) δ 163.6, 150.9, 134.8, 110.1, 87.0, 86.5, 71.3, 62.2, 51.0, 40.7, 40.2, 26.2, 24.8, 13.2. ESI-MS (*m*/*s*): 339.9 (calc. 340.2 [M + H^+^]). HRMS (*m*/*s*): 340.1616 [C_14_H_21_N_5_O_5_]H^+^ (calc. 340.1615).

*3-(5-Azidopentyl)-1-((2R,4S,5R)-4-hydroxy-5-(hydroxymethyl)tetrahydrofuran-2-yl)-5-methylpyrimidine-2,4(1H,3H)-dione* (**3d**). Compound **2d** (300 mg, 0.769 mmol) and NaN_3_ (500 mg, 7.69 mmol) were added into CH_3_CN (10 mL). The mixture was refluxed for 5 h and was followed by TLC. The solvent was evaporated under a reduced pressure and the crude product was purified by column chromatography with EtOAc to give **3d** as colorless oil (130 mg, yield 48%). ^1^H-NMR (400 MHz,CDCl_3_): δ 7.34 (d, *J* = 1.3 Hz, 1H), 6.18 (t, *J* = 6.9 Hz, 1H), 4.59 (dt, *J* = 6.8, 3.5 Hz, 1H), 4.01 (q, *J* = 3.2 Hz, 1H), 3.97–3.80 (m, 4H), 3.27 (t, *J* = 6.9 Hz, 2H), 2.76–2.21 (m, 4H), 1.92 (d, *J* = 1.2 Hz, 3H), 1.72–1.56 (m, 4H), 1.51–1.35 (m, 2H). ^13^C-NMR (100 MHz, CDCl3): δ 163.4, 150.6, 134.6, 110.0, 86.9, 86.0, 71.0, 62.0, 50.9, 40.9, 40.0, 28.1, 26.7, 23.7, 13.0. ESI-MS (*m*/*s*): 353.9 (calc. 353.4 [M + H^+^]). HRMS (*m*/*s*): 354.1774 [C_15_H_23_N_5_O_5_]H^+^ (calc. 354.1771).

*Di-tert-butyl-9-((1-(2-(3-((2R,4S,5R)-4-((tert-butyldimethylsilyl)oxy)-5-(((tert butyldimethylsilyl)oxy)methyl)tetrahydrofuran-2-yl)-5-methyl-2,6-dioxo-3,6-dihydropyrimidin-1(2H)-yl)ethyl)-1H-1,2,3-triazol-4-yl)methyl)-1,5,9-triazacyclododecane-1,5-dicarboxylate* (**5a**). Compound **3a** (400 mg, 0.74 mmol) was dissolved in THF (10 mL) and compound **4** (364 mg, 0.89 mmol) was added. After an addition of vitamin C (40 mg, 0.23 mmol), CuSO_4_·5H_2_O (15 mg, 0.06 mmol) was added under nitrogen. The reaction was stirred overnight at room temperature. The solvent was evaporated under a reduced pressure and the crude product was purified by column chromatography with CH_2_Cl_2_/EtOAc (*v*/*v* = 20:1) to give **5a** as white solid (500 mg, yield 71%). ^1^H-NMR (400 MHz, CDCl_3_): δ 7.49–7.41 (m, 2H), 6.25 (dd, *J* = 7.8, 5.8 Hz, 1H), 4.61 (t, *J* = 6.3 Hz, 2H), 4.45–4.31 (m, 3H), 3.91 (q, *J* = 2.6 Hz, 1H), 3.87–3.70 (m, 4H), 3.44–3.22 (m, 8H), 2.48–2.32 (m, 4H), 2.21 (ddd, *J* = 13.1, 5.8, 2.7 Hz, 1H), 1.96 (ddd, *J* = 13.5, 7.9, 6.0 Hz, 1H), 1.91–1.78 (m, 9H), 1.43 (s, 18H), 0.89 (d, *J* = 12.6 Hz, 18H), 0.09–0.06 (m, 12H). ^13^C-NMR (100 MHz, CDCl_3_): δ 162.9, 156.2, 150.4, 133.9, 122.7, 109.6, 87.7, 85.4, 80.0, 72.0, 62.8, 49.5, 47.2, 46.0, 45.4, 43.6, 41.3, 40.4, 28.4, 26.8, 26.1, 25.9, 25.7, 18.3, 17.9, 13.1, −4.7, −4.9, −5.4, −5.5. ESI-MS (*m*/*s*): 949.4 (calc. 949.6 [M + H^+^]). HRMS (*m*/*s*): 949.5972 [C_46_H_84_N_8_O_9_Si_2_]H^+^ (calc. 949.5972).

*Di-tert-butyl-9-((1-(3-(3-((2R,4S,5R)-4-hydroxy-5-(hydroxymethyl)tetrahydrofuran-2-yl)-5-methyl-2,6-dioxo-3,6-dihydropyrimidin-1(2H)-yl)propyl)-1H-1,2,3-triazol-4-yl)methyl)-1,5,9 triazacyclododecane-1,5-dicarboxylate* (**5b**). Compound **3b** (200 mg, 0.62 mmol) was dissolved in THF (10 mL) and compound **4** (251 mg, 0.62 mmol) was added. After an addition of vitamin C (40 mg, 0.23 mmol), CuSO_4_·5H_2_O (20 mg, 0.08 mmol) was added under nitrogen. The reaction was stirred overnight at room temperature. The solvent was evaporated under a reduced pressure and the crude product was purified by column chromatography with EtOAc/MeOH (*v*/*v* = 20:1) to give **5b** as white solid (270 mg, yield 60%). ^1^H-NMR (400 MHz, DMSO-*d*_6_): δ 8.02 (s, 1H), 7.77 (d, *J* = 1.4 Hz, 1H), 6.19 (t, *J* = 6.7 Hz, 1H), 5.23 (d, *J* = 4.2 Hz, 1H), 5.03 (t, *J*= 5.1 Hz, 1H), 4.36 (t, *J* = 7.1 Hz, 2H), 4.24 (p, *J* = 4.3 Hz, 1H), 3.83 (t, *J* = 7.2 Hz, 2H), 3.77 (q, *J* = 3.6 Hz, 1H), 3.69 (s, 2H), 3.65–3.51 (m, 2H), 3.24 (dt, *J* = 6.8, 3.6 Hz, 8H), 2.30 (t, *J* = 6.1 Hz, 4H), 2.15–2.01 (m, 4H), 1.87–1.69 (m, 9H), 1.38 (s, 18H). ^13^C-NMR (100 MHz, CDCl_3_): δ 163.6, 156., 150.9, 135.2, 123.1, 109.8, 87.4, 86.4, 79.6, 77.4, 70.9, 62.0, 49.6, 48.2, 46.8, 45.4, 43.8, 40.6, 38.5, 28.5, 28.3, 27.0, 26.1, 13.3. ESI-MS (*m*/*s*): 736.1 (calc. 736.4 [M + 2H^+^]). HRMS (*m*/*s*): 735.4396 [C_35_H_58_N_8_O_9_]H^+^ (calc. 735.4399).

*Di-tert-butyl-9-((1-(4-(3-((2R,4S,5R)-4-hydroxy-5-(hydroxymethyl)tetrahydrofuran-2-yl)-5-methyl-2,6-dioxo-3,6-dihydropyrimidin-1(2H)-yl)butyl)-1H-1,2,3-triazol-4-yl)methyl)-1,5,9-triazacyclododecane-1,5-dicarboxylate* (**5c**). Compound **3c** (400 mg, 1.18 mmol) was dissolved in THF (10 mL) and compound **4** (578 mg, 1.41 mmol) was added. After an addition of vitamin C (60 mg, 0.34 mmol), CuSO_4_·5H_2_O (20 mg, 0.06 mmol) was added under nitrogen. The reaction was stirred overnight at room temperature. The solvent was evaporated under a reduced pressure and the crude product was purified by column chromatography EtOAc/MeOH (*v*/*v* = 20:1) to give **5c** as white solid (500 mg, yield 57%). ^1^H-NMR (400 MHz, CDCl_3_): δ 7.61–7.53 (m, 2H), 6.27 (t, *J* = 6.7 Hz, 1H), 4.54 (dt, *J* = 6.6, 3.4 Hz, 1H), 4.40 (q, *J* = 7.0, 5.3 Hz, 2H), 4.04–3.91 (m, 3H), 3.91–3.72 (m, 4H), 3.39–3.23 (m, 8H), 2.50–2.20 (m, 6H), 2.02 (d, *J* = 1.8 Hz, 1H), 1.96–1.78 (m, 12H), 1.66–1.55 (m, 2H), 1.42 (s, 18H). ^13^C-NMR (100 MHz, CDCl_3_): δ 163.5, 156.4, 150.8, 142.7, 134.9, 123.1, 109.8, 87.3, 86.1, 79.5, 71.00, 62.0, 49.4, 46.2, 45.5, 43.8, 40.5, 30.0, 28.4, 27.5, 27.0, 25.8, 24.2, 13.2. ESI-MS (*m*/*s*): 749.3 (calc. 749.4 [M + H^+^]). HRMS (*m*/*s*): 749.4557 [C_36_H_60_N_8_O_9_]H^+^ (calc. 749.4556).

*Di-tert-butyl-9-((1-(5-(3-((2R,4S,5R)-4-hydroxy-5-(hydroxymethyl)tetrahydrofuran-2-yl)-5-methyl-2,6-dioxo-3,6-dihydropyrimidin-1(2H)-yl)pentyl)-1H-1,2,3-triazol-4-yl)methyl)-1,5,9-triazacyclododecane-1,5-dicarboxylate* (**5d**). Compound **3d** (300 mg, 0.849 mmol) was dissolved in THF (10 mL) and compound **4** (578 mg, 1.02 mmol)was added. After an addition of vitamin C (45 mg, 0.26 mmol), CuSO_4_·5H_2_O (15 mg, 0.08 mmol) was added under nitrogen. The reaction was stirred overnight at room temperature. The solvent was evaporated under a reduced pressure and the crude product was purified by column chromatography EtOAc/MeOH (*v*/*v* = 20:1) to give **5d** as white solid (552 mg, yield 62%). ^1^H-NMR (400 MHz, CDCl_3_): δ 7.58 (d, *J* = 1.4 Hz, 1H), 7.42 (s, 1H), 6.22 (t, *J* = 6.5 Hz, 1H), 4.55 (dt, *J* = 6.2, 4.1 Hz, 1H), 4.32 (t, *J* = 7.0 Hz, 2H), 4.06–3.98 (m, 2H), 3.93–3.73 (m, 6H), 3.31 (t, *J* = 6.7 Hz, 8H), 2.50–2.36 (m, 4H), 2.30 (m, 2H), 1.96–1.76 (m, 12H), 1.71–1.54 (m, 2H), 1.42 (s, 18H), 1.26–1.36 (m, 2H). ^13^C-NMR (100 MHz, CDCl_3_): δ 163.6, 156.5, 1501.0, 143.1, 135.0, 122.7, 109.9, 87.3, 86.5, 79.1, 70.9, 62.0, 60.5, 50.1, 49.5, 46.5, 45.5, 43.9, 40.7, 40.6, 29.7, 28.5, 26.7, 26.0, 23.6, 13.3. ESI-MS (*m*/*s*): 763.3 (calc. 763.5 [M + H^+^]). HRMS (*m*/*s*): 763.4716 [C_37_H_62_N_8_O_9_]H^+^ (calc. 763.4712).

*3-(2-(4-((1,5,9-Triazacyclododecan-1-yl)methyl)-1H-1,2,3-triazol-1-yl)ethyl)-1-((2R,4S,5R)-4-hydroxy-5-(hydroxymethyl)tetrahydrofuran-2-yl)-5-methylpyrimidine-2,4(1H,3H)-dione* (**6a**, **12N3-2-TdR**). Compound **5a** (500 mg, 0.53 mmol) was dissolved in ethanol (30 mL). After an addition of concentrate hydrochloric acid (1 mL), the reaction mixture was refluxed for 1 h. Solvent was removed under a reduced pressure. The mixture was recrystallized from ethanol (100 mL) to give **6a** as white solid (200 mg, yield 72.7%). ^1^H-NMR (400 MHz, D_2_O): δ 8.12 (s, 1H), 7.69 (d, *J* = 1.4 Hz, 1H), 6.25 (t, *J* = 6.7 Hz, 1H), 4.82–4.80 (m, 2H), 4.50 (q, *J* = 4.6 Hz, 1H), 4.45 (dd, *J* = 7.0, 4.9 Hz, 2H), 4.07 (dt, *J* = 5.2, 3.8 Hz, 1H), 4.03 (s, 2H), 3.73 (ddd, *J* = 42.3, 11.4, 2.6 Hz, 2H), 3.39 (dt, *J* = 12.3, 5.8 Hz, 8H), 2.91 (s, 4H), 2.43–2.31 (m, 4H), 2.18–2.08 (m, 4H), 1.90 (s, 3H). ^13^C-NMR (100 MHz, D_2_O): δ(ppm) 164.7, 151.2, 135.7, 128.1, 110.2, 86.6, 85.7, 70.3, 61.2, 57.4, 48.3, 47.3, 42.1, 41.3, 41.1, 38.7, 20.0, 17.9, 16.9, 12.3. ESI-MS (*m*/*s*): 521.1 (calc. 521.3 [M + H^+^]). HRMS (*m*/*s*): 521.3196 [C_24_H_40_N_8_O_5_]H^+^ (calc. 521.3194).

*3-(3-(4-((1,5,9-Triazacyclododecan-1-yl)methyl)-1H-1,2,3-triazol-1-yl)propyl)-1-((2R,4S,5R)-4-hydroxy-5-(hydroxymethyl)tetrahydrofuran-2-yl)-5-methylpyrimidine-2,4(1H,3H)-dione* (**6b**, **12N3-3-TdR**). Compound **5b** (270 mg, 0.37 mmol) was dissolved in ethanol (20 mL). After an addition of concentrate hydrochloric acid (1 mL), the reaction mixture was refluxed for 1 h. Solvent was removed under a reduced pressure. The mixture was recrystallized from ethanol (50 mL) to give **6b** as white solid (120 mg, yield 60.7%). ^1^H-NMR (400 MHz, D_2_O): δ 8.26 (s, 1H), 7.55 (d, *J* = 1.4 Hz, 1H), 6.17 (t, *J* = 6.7 Hz, 1H), 4.53–4.44 (m, 4H), 4.43–4.33 (m, 1H), 3.95 (dt, *J* = 5.2, 3.7 Hz, 1H), 3.87 (t, *J* = 6.9 Hz, 2H), 3.73 (ddd, *J* = 42.1, 11.4, 2.6 Hz, 2H), 3.46–3.28 (m, 12H), 2.34–2.17 (m, 10H), 1.83 (s, 3H). ^13^C-NMR(100 MHz, D_2_O): δ(ppm) 165.1, 151.3, 135.5, 127.4, 110.4, 86.5, 85.8, 70.4, 61.2, 57.4, 48.6, 48.5, 47.5, 42.1, 41.1, 38.7, 26.7, 20.0, 18.1, 16.9, 12.3. ESI-MS (*m*/*s*): 535.0 (calc. 535.3 [M + H^+^]). HRMS (*m*/*s*): 535.3349 [C_25_H_42_N_8_O_5_]H^+^ (calc. 535.3350).

*3-(4-(4-((1,5,9-Triazacyclododecan-1-yl)methyl)-1H-1,2,3-triazol-1-yl)butyl)-1-((2R,4S,5R)-4-hydroxy-5-(hydroxymethyl)tetrahydrofuran-2-yl)-5-methylpyrimidine-2,4(1H,3H)-dione* (**6c**, **12N3-4-TdR**). Compound **5c** (500 mg, 0.67 mmol) was dissolved in ethanol (20 mL). After an addition of concentrate hydrochloric acid (1 mL), the reaction mixture was refluxed for 1 h. Solvent was removed under a reduced pressure. The mixture was recrystallized from ethanol (50 mL) to give **6c** as white solid (216 mg, yield 58.9%). ^1^H-NMR (400 MHz, D_2_O): δ 8.22 (s, 1H), 7.57 (d, *J* = 1.3 Hz, 1H), 6.19 (t, *J* = 6.7 Hz, 1H), 4.51–4.31 (m, 5H), 3.94 (q, *J* = 4.1 Hz, 1H), 3.82 (t, *J* = 7.2 Hz, 2H), 3.79–3.64 (m, 2H), 3.45–3.25 (m, 12H), 2.34–2.14 (m, 8H), 1.94–1.73 (m, 5H), 1.49 (p, *J* = 7.6 Hz, 2H). ^13^C-NMR (100 MHz, D_2_O): δ (ppm) 165.3, 151.5, 135.5, 127.4, 110.5, 86.5, 85.8, 70.4, 61.2, 57.4, 50.1, 47.7, 42.3, 41.2, 40.7, 38.7, 26.7, 23.6, 20.0, 18.1, 16.9, 12.3. ESI-MS (*m*/*s*): 549.0 (calc. 549.0 [M + H^+^]). HRMS (*m*/*s*): 549.3505 [C_26_H_44_N_8_O_5_]H^+^ (calc. 549.3507).

*3-(5-(4-((1,5,9-Triazacyclododecan-1-yl)methyl)-1H-1,2,3-triazol-1-yl)pentyl)-1-((2R,4S,5R)-4-hydroxy-5-(hydroxymethyl)tetrahydrofuran-2-yl)-5-methylpyrimidine-2,4(1H,3H)-dione* (**6d**, **12N3-5-TdR**). Compound **5d** (552 mg, 0.73 mmol) was dissolved in ethanol (20 mL). After an addition of concentrate hydrochloric acid (1 mL), the reaction mixture was refluxed for 1 h. Solvent was removed under a reduced pressure. The mixture was recrystallized from ethanol (50 mL) to give **6d** as white solid (220 mg, yield 54%). ^1^H-NMR (400 MHz, D_2_O): δ 8.19 (s, 1H), 7.58 (s, 1H), 6.21 (t, *J* = 6.6 Hz, 1H), 4.49–4.32 (m, 5H), 3.95 (q, *J* = 4.3 Hz, 1H), 3.85–3.64 (m, 4H),3.45–3.19 (m, 12H), 2.42–2.08 (m, 8H), 1.95–1.76 (m, 5H), 1.52 (p, *J* = 7.6 Hz, 2H), 1.20 (p, *J* = 7.8 Hz, 2H). ^13^C-NMR (100 MHz, D_2_O): δ (ppm) 165.3, 151.5, 135.5, 127.1, 110.5, 86.5, 85.8, 70.4, 61.2, 57.4, 50.4, 48.1, 42.5, 41.3, 41.2, 38.7, 28.8, 25.6, 22.9, 19.9, 18.4, 16.8, 12.3. ESI-MS (*m*/*s*): 563.4 (calc. 563.4 [M + H^+^]). HRMS (*m*/*s*): 563.3662 [C_27_H_46_N_8_O_5_]H^+^ (calc. 563.3663).

### 3.3. Radiolabeling

The tricarbonyl technetium precursor was prepared according to the literature published by Alberto and his coworkers [24,34] with little modification. Namely, potassium sodium tartrate (15 mg), Na_2_CO_3_ (5 mg), and NaBH_4_ (10 mg) were added to a 10 mL glass vial. The vial was sealed and flushed with CO for 15 min, which is followed by the addition of 1 mL of saline containing [^99m^TcO_4_]^−^. The vial was heated at 80 °C for 30 min and then the [^99m^Tc(CO)_3_(H_2_O)_3_]^+^ precursor was prepared. After being cooled to the room temperature, 1.0 mol/L HCl was added to adjust the pH to approximately 7. Then, 0.5 mL of phosphate-buffer (0.2 mol/L, pH = 7.2) containing 1.0 mg of ligand 6 was added, and the reaction mixture was heated at 100 °C for 30 min under nitrogen (Scheme 2). After being cooled to the room temperature, the radiochemical purity (RCP) of the complexes was evaluated by HPLC. Water (containing 0.1% TFA) (A) and acetonitrile (containing 0.1% TFA) were used as the mobile phase. The gradient elution technique was adopted for the preparation: 0 min 10% B, 2 min 10% B, 10 min 90% B, 18 min 10% B.

### 3.4. Stability Studies

The complexes were incubated in saline at room temperature for 6 h, and then the stabilities of the complexes were measured by HPLC. To evaluate the serum stability of the complexes, 0.1 mL (3.7 MBq) of **7a**, **7b**, **7c** and **7d** were incubated in 0.5 mL of mouse serum at 37 °C for 6 h, and then the RCP of the complex was measured by HPLC after removing the proteins.

### 3.5. Octanol/Water Partition Coefficient

The partition coefficient was measured by mixing the complex with an equal volume of 1-octanol and phosphate buffer (0.025 mol/L, pH 7.4) in a 10 mL centrifugal tube. The mixture was vigorously vortexed for 5 min, and then centrifuged at 14000 rpm for another 5 min. Three samples (100 μL) in triplets from 1-octanol and phosphate buffer were pipetted and measured in a well γ-counter. The partition coefficient was calculated using the following equation: *P* = (counts per minute in octanol/counts per minute in buffer). Usually, Log *P* was expressed as the final partition coefficient value.

### 3.6. In Vitro Cell Experiments

Murine sarcoma S180 cell lines were extracted from tumor-bearing mice. The cells were washed three times by saline. S180 cells were grown in DMEM (Dulbecco Modified Eagle Medium) medium containing 10% (*v*/*v*) of fetal bovine serum at a cell concentration of 2 × 10^4^ cells/mL. One culture flask containing 1.0 mL cell suspension was added to 0.074 MBq of **7d**, and the others were added to 0.074 MBq of **7d** and 0.1 mL of saline which contained different amount of thymidine. After incubation for 2 h, the cells were centrifuged at 10,000 rpm for 5 min for pellet formation. The cells after pelleting were washed three times with phosphate-buffer (0.2 mol/L, pH = 7.2). Each supernatant was removed for counting purposes. The percentage of cell uptake is calculated as residue counts/the total counts × 100%. The studies were measured five times. The final results were expressed as an average of five measurements plus the standard deviation.

### 3.7. Biodistribution Study

Animal studies were carried out in compliance with the Regulations on Laboratory Animals of Beijing Municipality and the guidelines of the Ethics Committee of Beijing Normal University. The experiments were approved by the Ethics Committee of Beijing Normal University. The biodistribution of **7a**–**d** was evaluated in Kunming male mice (18–22 g) bearing S180 tumors. The complex (0.1 mL, 3.7 × 10^5^ Bq) was injected into the mice via a tail vein. At 0.5, 2, 4 and 6 h post-injection, the mice were sacrificed by neck dislocation. The tumors and other interesting organs including blood were collected, weighed and measured for radioactivity. The final results were expressed as the percent uptake of injected dose per gram of tissue (%ID/g).

## 4. Conclusions

In the present study, four novel thymidine analogs were synthesized via “click reaction” route, and their ^99m^Tc(CO)_3_ complexes were successfully prepared in high yields through a ligand-exchange reaction. They were hydrophilic and stable *in vitro*. The preliminary *in vivo* studies showed that all of them had a relative high tumor uptake and tumor-to-muscle ratio. Further studies should be conducted to evaluate the possibilities of these ^99m^Tc(CO)_3_ complexes as radiotracers for tumor proliferation imaging.

## Figures and Tables

**Table 1:** Biodistribution of **7a** in mice bearing S180 tumor (mean ± SD, *n* = 4, %ID/g).

Tissue	30 min	120 min	240 min	360 min
Heart	2.48 ± 1.15	1.65 ± 1.12	1.26 ± 0.48	0.74 ± 0.29
Liver	9.94 ± 2.33	9.12 ± 2.09	8.10 ± 0.54	7.98 ± 1.00
Lung	2.92 ± 0.63	1.41 ± 0.33	0.92 ± 0.08	0.85 ± 0.15
Kidney	14.12 ± 1.56	9.90 ± 1.24	7.71 ± 1.04	8.35 ± 0.91
Spleen	1.77 ± 0.68	1.58 ± 0.90	0.86 ± 0.07	0.83 ± 0.09
Stomach	2.06 ± 0.69	1.57 ± 1.07	1.35 ± 0.48	1.31 ± 0.20
Bone	3.41 ± 0.85	2.73 ± 1.22	2.47 ± 0.30	2.14 ± 0.76
Muscle	1.65 ± 0.80	0.67 ± 0.11	0.88 ± 0.32	0.59 ± 0.24
Intestine	6.20 ± 1.07	4.06 ± 1.43	2.57 ± 0.80	2.62 ± 1.50
Tumor	2.16 ± 0.59	0.96 ± 0.45	0.57 ± 0.15	0.55 ± 0.14
Blood	2.50 ± 0.41	1.06 ± 0.48	0.54 ± 0.05	0.43 ± 0.09
Thyroid (ID%)	0.14 ± 0.01	0.11 ± 0.05	0.14 ± 0.05	0.05 ± 0.03
Tumor/Blood	0.86	0.90	2.02	1.29
Tumor/Muscle	1.31	1.44	1.23	0.94

**Table 2:** Biodistribution of **7b** in mice bearing S180 tumor (mean ± SD, *n* = 4, %ID/g).

Tissue	30 min	120 min	240 min	360 min
Heart	1.08 ± 0.24	0.77 ± 0.17	0.46 ± 0.06	0.51 ± 0.11
Liver	17.49 ± 4.40	9.22 ± 1.19	8.52 ± 0.32	7.13 ± 0.90
Lung	2.25 ± 0.35	1.15 ± 0.14	0.95 ± 0.11	0.79 ± 0.13
Kidney	14.54 ± 2.24	6.52 ± 0.69	5.18 ± 0.63	4.58 ± 0.85
Spleen	1.12 ± 0.17	1.58 ± 0.09	0.61 ± 0.05	0.45 ± 0.12
Stomach	1.04 ± 0.27	1.00 ± 0.41	0.87 ± 0.27	0.94 ± 0.33
Bone	1.77 ± 0.24	1.26 ± 0.93	0.84 ± 0.31	0.66 ± 0.32
Muscle	0.72 ± 0.10	0.29 ± 0.06	0.29 ± 0.05	0.25 ± 0.13
Intestine	5.21 ± 0.19	3.12 ± 1.01	2.50 ± 0.83	2.27 ± 0.36
Tumor	1.28 ± 0.46	0.70 ± 0.16	0.63 ± 0.15	0.46 ± 0.08
Blood	1.87 ± 0.28	0.92 ± 0.14	0.67 ± 0.06	0.65 ± 0.29
Thyroid (ID%)	0.07 ± 0.03	0.05 ± 0.02	0.04 ± 0.01	0.02 ± 0.01
Tumor/Blood	0.68	0.76	0.95	0.71
Tumor/Muscle	1.79	2.39	2.22	1.83

**Table 3:** Biodistribution of **7c** in mice bearing S180 tumor (mean ± SD, *n* = 4, %ID/g).

Tissue	30 min	120 min	240 min	360 min
Heart	0.72 ± 0.14	0.48 ± 0.20	0.43 ± 0.20	0.41 ± 0.11
Liver	8.70 ± 1.70	7.54 ± 0.95	9.58 ± 0.57	7.08 ± 1.52
Lung	2.64 ± 1.23	1.07 ± 0.14	1.03 ± 0.44	1.13 ± 0.42
Kidney	15.79 ± 1.30	12.23 ± 2.03	9.84 ± 1.77	8.48 ± 0.78
Spleen	0.85 ± 0.07	1.58 ± 0.14	0.64 ± 0.16	0.61 ± 0.18
Stomach	0.63 ± 0.34	0.32 ± 0.07	0.24 ± 0.05	0.36 ± 0.07
Bone	1.09 ± 0.33	0.79 ± 0.29	0.57 ± 0.26	0.97 ± 0.46
Muscle	0.47 ± 0.18	0.30 ± 0.07	0.19 ± 0.06	0.24 ± 0.11
Intestine	2.38 ± 0.30	1.10 ± 0.32	0.95 ± 0.55	0.98 ± 0.35
Tumor	1.57 ± 0.31	0.63 ± 0.18	0.47 ± 0.11	0.40 ± 0.06
Blood	1.83 ± 0.32	0.60 ± 0.09	0.53 ± 0.10	0.44 ± 0.03
Thyroid (ID%)	0.07 ± 0.03	0.05 ± 0.02	0.04 ± 0.01	0.02 ± 0.01
Tumor/Blood	0.86	1.06	0.90	0.92
Tumor/Muscle	3.36	2.11	2.49	1.70

**Table 4:** Biodistribution of **7d** in mice bearing S180 tumor (mean ± SD, *n* = 4, %ID/g).

Tissue	30 min	120 min	240 min	360 min
Heart	1.12 ± 0.27	0.63 ± 0.22	0.53 ± 0.14	0.36 ± 0.04
Liver	14.21 ± 2.40	11.70 ± 4.83	8.27 ± 2.09	8.86 ± 2.60
Lung	2.72 ± 0.56	1.39 ± 0.28	1.21 ± 0.29	0.96 ± 0.33
Kidney	11.40 ± 2.63	6.79 ± 1.20	5.24 ± 1.11	4.38 ± 0.53
Spleen	1.46 ± 0.47	1.58 ± 0.44	0.77 ± 0.13	0.65 ± 0.12
Stomach	1.24 ± 0.62	0.31 ± 0.12	0.26 ± 0.08	0.28 ± 0.05
Bone	2.40 ± 0.59	0.88 ± 0.31	0.69 ± 0.22	0.41 ± 0.18
Muscle	0.53 ± 0.10	0.22 ± 0.05	0.20 ± 0.08	0.25 ± 0.10
Intestine	4.04 ± 0.79	1.31 ± 0.42	0.80 ± 0.30	0.66 ± 0.11
Tumor	1.90 ± 0.58	0.63 ± 0.14	0.33 ± 0.05	0.36 ± 0.16
Blood	1.75 ± 0.23	0.67 ± 0.08	0.52 ± 0.08	0.47 ± 0.05
Thyroid (ID%)	0.07 ± 0.03	0.05 ± 0.02	0.04 ± 0.01	0.09 ± 0.01
Tumor/Blood	1.08	0.95	0.64	0.77
Tumor/Muscle	3.56	2.88	1.66	1.48

## Supplementary Materials

Supplementary materials can be accessed at: http://www.mdpi.com/1420-3049/21/4/510/s1.
