# Supplementary material for: Synthesis and Biological Evaluation of Novel 99mTc(CO)3-Labeled Thymidine Analogs as Potential Probes for Tumor Proliferation Imaging
Source: Molecules. 2016 Apr 19;21(4):510. doi: 10.3390/molecules21040510 (PMC6274394; doi:10.3390/molecules21040510)
Supplement: Supplementary file 1 [file molecules-21-00510-s001.pdf]

Supplementary Materials: Synthesis and Biological Evaluation of Novel $^{99m}Tc(CO)_3$-Labeled Thymidine Analogs as Potential Probes for Tumor Proliferation Imaging

Xiaojiang Duan, Teli Liu, Yichun Zhang and Junbo Zhang

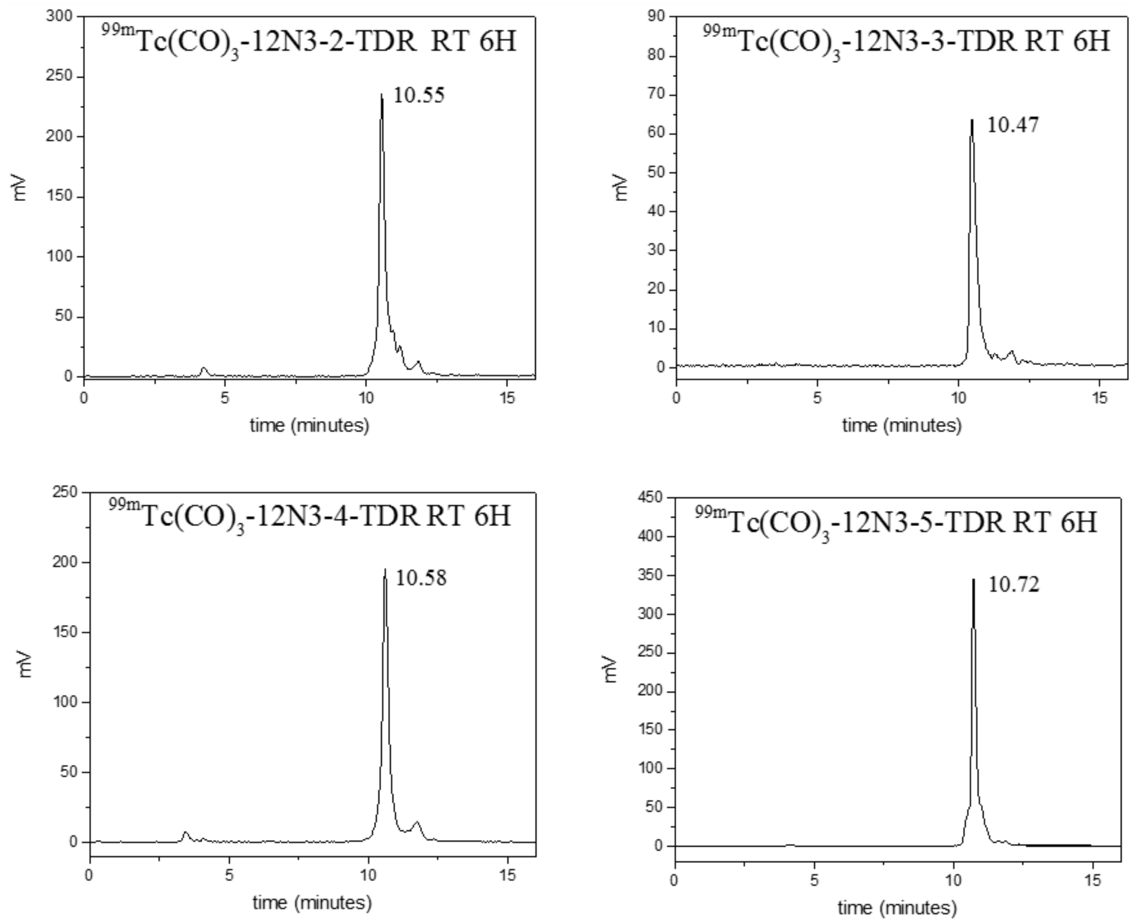

Figure S1. HPLC chromatograms of **7a**, **7b**, **7c** and **7d** at room temperature for 6 h.

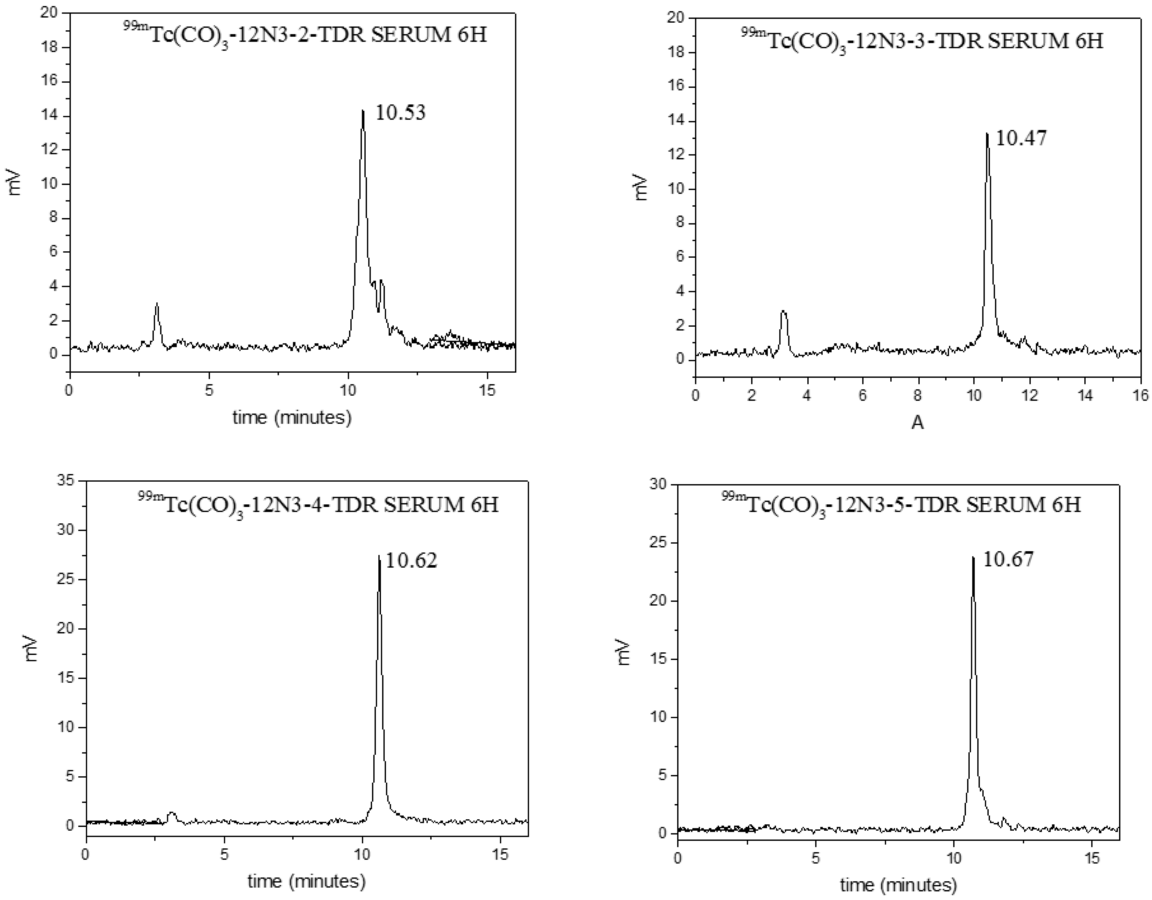

Figure S2. HPLC chromatograms of **7a**, **7b**, **7c** and **7d** in mouse serum at 37 °C for 6 h.
